# The learning performance and behavior of school children during the COVID-19 pandemic school closures: from the perspectives of parents and teachers

**DOI:** 10.1590/2317-1782/20232022037en

**Published:** 2023-08-04

**Authors:** Greicyane Marcos de Castro, Vanessa de Oliveira Martins-Reis, Letícia Correa Celeste

**Affiliations:** 1 Universidade de Brasília - UNB - Brasília (DF), Brasil.

**Keywords:** COVID-19, Speech, Language and Hearing Sciences, Perception, Child, Teaching, Social Vulnerability, Mental Health, Risk, Learning

## Abstract

**Purpose:**

To verify the association between caregivers' and teachers' perceptions of the changes imposed by social isolation and the impact on students' learning.

**Methods:**

This is an analytical observational and longitudinal study, with the participation of 19 caregivers (family members) of 2^nd^-grade students from a public financing school and their respective teachers. The caregivers were submitted to interviews by means of the questionnaires: Abilities and Difficulties Questionnaire, Family Environment Resources Inventory and COVID-19 monitoring questionnaire that checks the behavior of schoolchildren and families. The collection took place in two moments by telephone call: the first moment (M1) was started in June 2020, and the second, (M2) in December 2020. The progress reports prepared by the regular teachers were received in January 2021.

**Results:**

A negative implication was observed in the change of routine and an impact on the parents' lives. In parents' perceptions, significant and negative issues were also present in children's mental health, such as changes in routine. Teacher evaluation reports showed a similar pattern, with the vast majority of them lacking information about learning performance.

**Conclusion:**

This study pointed out the importance of accompanying schoolchildren and families in a period of social withdrawal, in order to guide and intervene together with parents and teachers.

## INTRODUCTION

Social distancing was introduced in various Brazilian states as a preventive measure to help combat the spread of COVID-19, this included school closures. Governmental (Decree nº 40.550 of March 23, 2020 of Brazilian’s Federal District). School plays a crucial role in the development of children and with their closures lessons had to be adapted to an online format so that there was no interruption to classes. With this change came the need to adopt strategies that would help to reduce the impact caused by the suspension of in-person lessons^(1)^.

Digital education in Brazil was already facing many challenges and impasses, such as difficulties to access the internet in the classroom, problems to train teachers in activity planning, among others^(2)^. Considering such difficulties and limitations it is important to reflect on the impact caused to the process of learning to read and write during this period of online teaching, especially to children at the beginning of elementary school.

When comparing in-person teaching with online teaching it is necessary to take into account the issues and challenges faced, whether from the viewpoint of teachers, children, families or the technologies employed. The teacher has to adapt and learn how to use these new technologies in his or her professional daily life so that there can be an interaction with the students and to prepare videos and exercises among other activities. For the families the main challenge is the availability and awareness of those responsible, to be able to follow the lessons and or explain the content to children. Very often the parents or guardian did not have a sufficient level of education to do this. Children need motivation to maintain their attention and study pace, and to achieve this previous development of skills and academic content is essential.

Technology includes access and the ability to use equipment and also connection quality, there is a strong link between these issues and socioeconomic status, since families with low socioeconomic status do not have sufficient access to technology and those responsible tend to have a poorer level of schooling^(3)^. Such questions lead us to ask whether the conditions of access that encompass the online schooling process are being sufficiently monitored.

It is a well-known fact that cognitive and linguistic development is associated with socioeconomic status, and children from low income families have a poorer academic performance^(4)^, therefore it is important to accompany students in vulnerable situations, since access to schooling sometimes has further to go, becoming even more important in the context of online teaching and the return to in-person teaching. When one knows how the pace of development strategies can be employed to minimize the impact caused to families.

Studies show that mental health and family resources are also associated with the academic performance of school children^(5-7)^. A study carried out in São Paulo with students from the 1st to 5th years showed that students who had poorer school performance in the opinion of those responsible are the ones with the greatest problems related to behavior and hyperactivity^(8)^.

In addition to the impact on mental health, the family environment also positively influences school performance^(9)^, when there is a family interaction with pre-established routines and greater access to extracurricular activities^(10)^, the encouragement of reading and monitoring of school activities^(11)^ and the family-school relationship^(12)^.

During social distancing the family-school relationship became distant in the context of the conventional classroom and there was a greater need for family participation and support during this time^(13)^. In addition children were socially isolated and missed out on opportunities for leisure and socializing with people outside of their families. Understanding how these families experienced and are experiencing this period of confinement is a way to help them to understand how to face such difficulties. The objective of this study was to analyze the changes brought about by social isolation and the impact on the learning development of students in the second year of a public elementary school from the viewpoint of parents and teachers.

## METHODS

This is an analytical and longitudinal observational study approved by the Research Ethics Committee of the institution under protocol number 3,906,514. All guardians signed a consent form (FICT). Those who participated in the study were parents or guardians made up of the closest family members responsible for the children, and the teachers of 19 students from the 2nd year of an elementary school. Data collection was carried out by phone survey and parents and guardians who agreed to participate were interviewed by telephone and the interviewer read out the consent form and the interviewees responded by giving their names and agreement as requested by the ethics committee and each interview was recorded.

This study is part of a larger project based on the Intervention Response Model (RTI) that began in February 2020 in-person. With the closure of schools, only children enrolled in classes of the second year of a public elementary school in the city of Brasília who had a signed consent form before the school closures were included in this study. Childrens´ parents who did not take part in the two interviews were excluded.

For data collection, the following instruments as answered by the (family members) were used:

- The Abilities and Difficulties Questionnaire (SDQ-Por)7 is an instrument used internationally to detect problems related to children's mental health, It consists of 25 items, 10 of which are about capabilities, 14 about difficulties and one neutral item, It is divided into five sub-scales each with five statements namely, emotional symptoms, conduct problems, hyperactivity, peer relationship problems and prosocial behavior. The questionnaire's score followed the proposal of the instrument itself, with the classification of the score of each scale and the total score as normal, borderline and altered.
- The Inventory of Family Environment Resources (RAF)^(14)^ is a script with closed-ended questions that makes it possible to survey the resources of the family environment that can contribute to school performance during the elementary school years. It consists of 10 questions divided into three domains, proximal processes (walks and trips, interaction with parents, access to toys, books, newspapers and magazines, adequate occupation of time), stability in family life (child participation in household chores, family routine) and family-school relationship (parent participation in school life and meetings).
- The COVID-19 Monitoring Questionnaire. Specially prepared for this study has 18 questions about the behavior of children and families as detailed below. The suspension of classes due to social isolation, family income during this period, how the child dealt with social isolation and information about preventative measures, and the child's routine and behavior during isolation. For each question a Likert scale of up to seven points was used. For the analysis of each variable, the Likert scale score of each item was added, with the following maximum total scores; parents explained preventative measures to schoolchildren 16 points, schoolchildren understood social distancing 12 points, knew that measures had to be taken 8 points, took or accepted to take preventive measures 16 points, leisure activities at home 10 points, impact of school suspension on parents' lives 12 points, changes in students' behavior 40 points, and changes in play 54 points. The closer to the maximum score, more positive the behavior.

End of school year reports. The children's progress was analyzed by reading the end of the school year reports for 2020 as prepared by the teachers.

Data collection was carried out in three steps, the first two being interviews with the guardians or parents of the children, and the third reception of the progress reports. The interviews were carried out by phone by the first interviewer and questions were asked from the first questionnaires as described above. The first moment (M1) started in June 2020, and the second moment (M2) in December 2020. The progress reports prepared by the teachers were received in January 2021. It is worth mentioning that school activities were resumed in an online format on the 13^th^ of July 2020 in public schools. During the previous months (April and June), a period in which there was no school, strategies were drawn up and improved on, to involve student through printed activities and an online platform.

The data was submitted for descriptive and inferential statistical analysis with a significance level of 5%. The analyzes were performed using the IBM SPSS statistical platform (Statistical Package for Social Sciences) version 21.0. The tests used to compare the two instances were, the Mcnemar Bowker, to measure the behavior at moment 1 (M1) and moment 2 (M2) of the same sample for dependent qualitative variables, and the Wilcoxon Rank Test for dependent quantitative variables . For correlation analysis the Spearman test was used for a non-normal distribution.

## RESULTS

Data analysis showed that more than 80% of respondents were mothers/fathers or grandmothers of the students. Regarding the suspension of classes, 94.7% agreed with the government's decision to suspend in-person classes as a coping measure imposed by COVID-19 and 68.4% agreed or strongly agreed to the suspension of classes for the same reasons mentioned above.

The data collected from the COVID-19 Monitoring Questionnaire did not generate the same variables in M1 and M2. For M2, the following variables were collected and compared, family income, access to emergency assistance, changes in the child's behavior, access to remote classes and hours of study. Table 1 shows the results of the descriptive statistics of the score obtained in each variable of the COVID-19 Monitoring Questionnaire in M1 and M2. In M2, only the variable change in the child's behavior was generated again, but no change was found in the score from M1 to M2 (p=0.536). To analyze the RAF in M1 and M2, we compared the following variables, number of toys, collection of school books, books on different topics, general outings, outings to special places, extracurricular activities, family-school relationship, routine, stability in family life, playtime outside, reading at home, talking about school and television topics. Statistically significant differences were found between the two moments only for the variables, amount of toys, general outings, outings to special places and family-school relationship (p-value<0.05) (Table 2).

**Table 1:** Result of the descriptive statistics of the COVID-19 Monitoring Questionnaire score

	M1			M2	
	f(%)			f(%)	
Access to emergency aid (Yes)	9 (47.39)		7 (36.84)		
Decreased income (Yes)	12 (63.2)		9 (47.4)		
Online class (No)	14 (74)		0 (0.00)		
How many hours per day spent with school activities					
0h	3 (15.8)			1 (5.3)	
up to 1h	12 (63.16)			9 (47.37)	
more than 1h	4 (21.05)			9 (47.37)	
	**Average**	**Median**	**SD**	**Minimum**	**Maximum**
Child understands social distancing	7.58	8.00	2.795	1	12
Family explained about preventative measures	15.26	16.00	1.522	10	16
Child knows the measures that must be adopted	6.95	8.00	1.433	3	8
Child takes or agrees to take preventive measures	13.26	14.00	2.306	8	16
Leisure at home with the child	6.42	6.00	2.090	1	9
Impact of the suspension of classes on parents' routine	14.95	15.00	4.478	4	26
Behavioral changes	48.63	49.00	5.540	37	57
Changes in play patterns	27.84	27.00	3.760	20	37

Data were expressed as frequency (f) and percentage (%)

**Caption:** M1 = Moment 1; M2 = Moment 2; SD = Standard deviation

**Table 2:** Results obtained in RAF in M1 and M2

		**Median**	**Maximum**	**Minimum**	**p-value** 1
Number of toys	M1	12	6	18	**0.017** *
	M2	15	9	17	
School book collection	M1	6	3	7	0.361
	M2	6	2	7	
Books on different topics	M1	5	2	6	0.361
	M2	5	1	6	
Number of outings	M1	4	0	4	0.020*
	M2	2	0	4	
Outings to special places	M1	6	1	9	0.003*
	M2	2	0	11	
Extracurricular activities	M1	1	0	6	0.065
	M2	1	0	1	
Family-school relationship	M1	8	4	8	<0.001*
	M2	10	5	10	
Routine	M1	11	0	16	0.739
	M2	12	6	16	
Stability in family life	M1	5	2	6	0.67
	M2	6	3	6	
		**F**	**%**		**p-value** 2
Playing outside	M1	10	52.63		1
	M2	11	57.89		
Reading at home	M1	10	52.63		0.289
	M2	14	73.68		
talking about school	M1	19	100.00		#
	M2	18	94.74		
Talking about television topics	M1	16	84.21		1
	M2	16	84.21		

* p<0.05

1 Mann-Whitney test

2 McNemar-Bowker test

Data were expressed as frequency (f) and percentage (%)

**Caption:** M1 = Moment 1; M2 = Moment 2; RAF = Inventory of Family Environment Resources; # = Could not calculate

Table 3 does not indicate a statistically significant difference for the SDQ-Por between M1 and M2 (p-value >0.05). The high number of children with altered and borderline scores in the total score stands out, and in some subscales they show difficulties for children in the items studied.

**Table 3:** Analysis of the Subscales of the Abilities and Difficulties Questionnaire (SDQ- Por) - M1 and M2 of the collection

		**Moment 1**	**Moment 2**
		**f (%)**	**f** **(%)**
Emotional problems	Normal	9 (47.4)	9 (47.4)
	Borderline	4 (21.0)	2 (10.5)
	Changed	7 (36.8)	8 (42.1)
Behavioral problems	Normal	12 (63.2)	15 (78.9)
	Borderline	3 (15.8)	1 (5.3)
	Changed	4 (21.0)	3 (15.8)
hyperactivity	Normal	13 (68.4)	15 (78.9)
	Borderline	1 (5.3)	0
	Changed	5 (26.3)	4 (21.0)
Problems with peers	Normal	13 (68.4)	14 (73.7)
	Borderline	3 (15.8)	2 (10.5)
	Changed	3 (15.8)	3 (15.8)
Prosocial behavior	Normal	19 (100)	19 (100)
	Borderline	0	0
	Changed	0	0
Impact	Normal	13 (68.4)	13 (68.4)
	Borderline	1 (5.3)	3 (15.8)
	Changed	5 (26.3)	4 (21.0)
Total	Normal	0	1 (5.3)
	Borderline	2 (10.5)	6 (31.6)
	Changed	17 (89.5)	12 (63.2)

McNemar-Bowker test, significance level at 5%

Data were expressed as frequency (f) and percentage (%)

**Caption:** M1 = Moment 1; M2 = Moment 2

**Figure 1:**
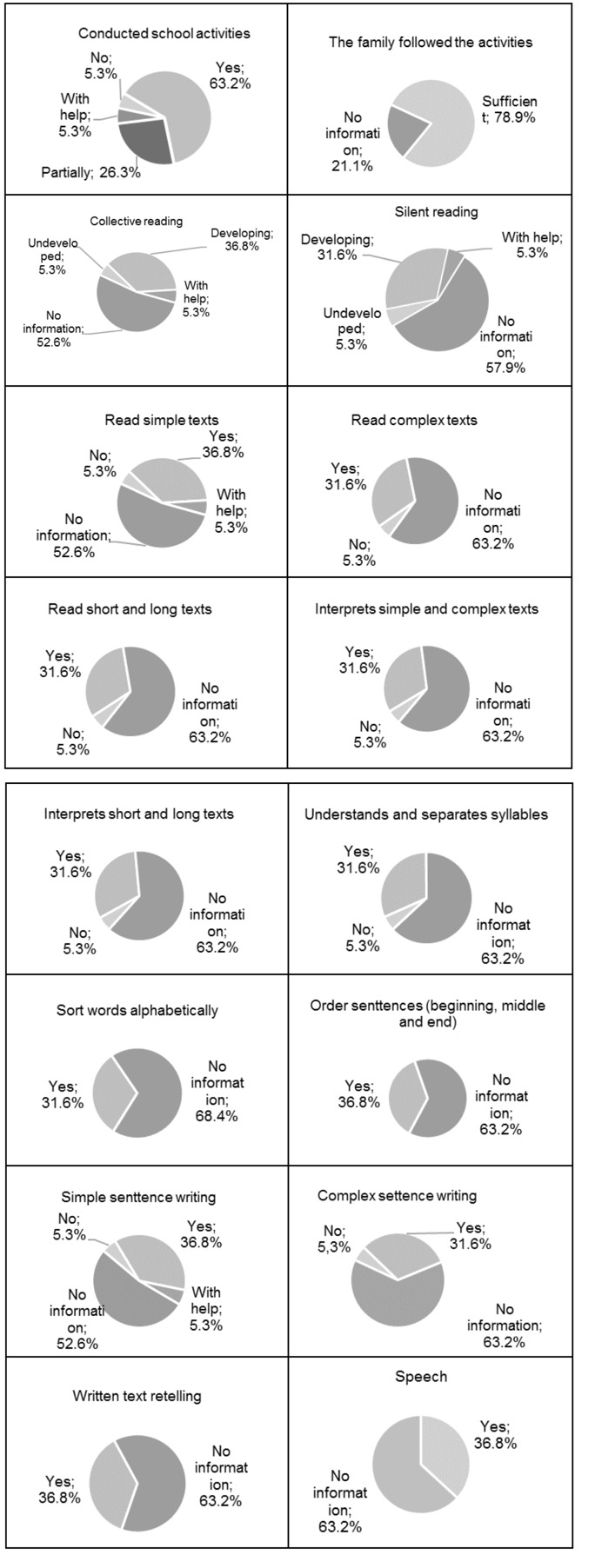
Teacher evaluation report at the end of the 2020 semester

Due to the difficulties faced by teachers in assessing the children's progress, online school reports show us a similar pattern, with the vast majority lacking information (with a little more than 60% of the sample missing information). For the analysis of the reports, we sought to identify the following elements about the children's development, carried out school activities, the family followed the activities, collective reading, silent reading, reading and interpretation of simple and complex texts, reading and interpretation of short and long texts, comprehension and syllable separation, ordering of words and phrases, writing simple and complex sentences, retelling of written text, and oral speech (Figure 1). It was found that 63.2% of the students were able to carry out their activities and 78.9% of the families monitored the activities, and only 5.3% did not carry out the activities that were given.

The teachers' evaluations showed us that 36.8% of the 19 students were developing collective reading and 31.6%, silent reading. 36.8% read simple texts and 31.6% read complex, short and long texts and interpreted simple and complex, short and long texts (Figure 1).

Regarding writing, 36.8% could write simple sentences and 31.6% could write complex sentences. Only 31.6% could order words in alphabetical order, and 36.8% of students could order sentences (beginning, middle and end). 31.6% understood and could separate syllables. 36.8% of students could retell written texts . In the oral speech performed by the students the percentage was 36.8%. The psychogenesis levels evaluated by the teachers showed that 5.3% of the students were in the pre-syllabic level, 15.8% in the alphabetic II level and 15.8% in the alphabetic III level(Figure 1). Other teachers did not contribute to the report.

A crossing between the RAF and the COVID-19 Monitoring Questionnaire was performed at moments M1 and M2. The Spearman correlation was used for the analysis. Because there are many variables we chose to present only statistically significant results. According to the results presented in Table 4, children who had more toys in M2 and took more walks in M1 understood social distancing better. Explaining preventative measures to children was more evident in the routine at M1. The stability of family life in M2 made the child aware of what preventive measures should be taken. Schoolchildren who complied with preventive measures had a reduction in general and special outings in M2. During leisure time, it was found that the students had a collection of books M1 and books with different themes M1. In M2, the decrease in extracurricular activities in the RAF impacted the parents' routines.

**Table 4:** Crossing between the questionnaires (RAF and Quest. COVID-19) at M1 and M2 of the collection

		Understood social distancing	Explained about preventive measures	Know what measures should be taken	Takes or accepts to take preventive measures	Leisure	Parents impacted
Number of toys - M2	ρ	0.614**					
	p-value	0.005					
General outings - M1	ρ	0.510*					
	p-value	0.026					
Routine - M1	ρ		0.571*				
	p-value		0.011				
Book Collections - M1	ρ					0.522*	
	p-value					0.022	
Books on various topics - M1	ρ					0.522*	
	p-value					0.022	
Stability in family life M2	ρ			0.579**			
	p-value			0.009			
General outings - M2	ρ				-0.485*		
	p-valor				0.035		
Outings to special places - M2	ρ				-0.548*		
	p-valor				0.015		
Extracurricular Activities - M2	ρ						0.515*
	p-valor						0.024

Spearman correlation coefficient test (〉), significance level at 5%

** p<0.01;

* p<0.05

**Caption:** M1 = Moment 1; M2 = Moment 2; RAF = Inventory of Family Environment Resources; # = Could not calculate

## DISCUSSION

The purpose of this study was to verify the association between the changes imposed by social distancing, the learning development and the behavior of students. We present the results of 19 children from the second year of an Elementary School from the perspective of their parents and teachers as collected in June and December of 2020.

The students were evaluated through questionnaires (parents' perspective) about the situation of school closures, the period of social isolation and the teachers' progress reports.

The data shows that the families agreed with the suspensions of classes due to the spread of the virus, but not everyone liked it. Similar findings were found in another survey carried out in the Czech Republic where despite a lack of technology and the difficulties in teaching school activities at home, parents still preferred to maintain social distancing and online teaching due to COVID-19^(15)^. Although the study deals with young people, the similarity with the present study is due to the contextual analysis of the perspective of the parents.

Most families did not have access to the emergency aid program in M2. It´s a well-known fact that a decrease in income is an aggravating factor in families and this was already being discussed well before the period of social isolation, however during the pandemic it was worse with an even greater impact with results dependent on the length of social isolation^(16)^.

Regarding how children coped with social isolation, most schoolchildren understood social distancing because parents were careful to explain about preventive measures that should be taken and when necessary. The influence of parents in health education is very important because children feel more comfortable and confident in the home environment^(17)^.

There were significant changes in leisure and play, children's behavior and parents' lives after the school closures^(18)^, however results did not show this. Because the beginning of data collection took place two months after the school closures, it could be concluded that families were already adapting to the changes imposed by the pandemic.

Classes as authorized by the Municipal Secretary of Education (SEE) started on The 10^th^ of February 2020, however they were suspended on the 12^th^ of March 2020 due to the COVID-19 pandemic. Online teaching started at SEE on the 13^th^ of July 2020 and some difficulties were found in accessing the classes at M1, with more than 70% of students without any type of access. However in the M2 of the collection, all students already had access to classes, with classes between one and six times a week and each class containing one hour or more of school activities.

It is important to emphasize that even though the students did not have access to classes at M1, their parents/guardians were concerned enough to dedicate a few hours to school activities with books and others materials of choice. Similar data can be found in terms of both study hours and parents/guardians´ dedication to school assignments in a survey carried out in the Czech Republic. This study (N=9,810) surveyed data from 72% of students who dedicated between 2-4 hours of study per day, and 66% of their parents/guardians helped during half of this time with school activities^(15)^_._

During social distancing, schoolchildren spent more time at home than usual. Regarding the resources of the family environment, the variable playing on the street did not change in both moments, but the amount of toys in M2 was greater, this may be because parents bought more toys to help improve the playtime of children, bringing a certain degree of comfort during self isolation^(19)^. However, the results show us that when it came to books no differences were found between M1 and M2, in fact book collection decreased in M2.

We can speculate that this decrease, could have been because of free access to virtual books, which were more accessible and accompanied by national campaigns or donations. However despite there being different studies on early childhood education during the pandemic^(20)^, we did not find academic articles that focused on the variation of children's books available during the pandemic in Brazil. Despite not being a specific objective of this study, the limitation that it was not possible to verify the variability of books during the period should be taken into account, data that is needed by the instruments used.

During social isolation family outings decreased considerably which shows the extent of caution that families took during social distancing in Brazil. The decrease in extracurricular activities during the pandemic had a negative effect because they are particularly important for children to help build relationships between peers and for socialization. Playing is one of the activities that most helps children in the process of affectivity, development and socialization^(20)^.

The school has always sought to involve children´s families in the students' learning process and the family is the first to start this process. Trust and commitment are part of the family school relationship and need to be in harmony to guarantee continued and effective education. In this study, we saw that families improved this relationship and they needed to have better contact with the school because they often accompanied their children during online classes^(21)^. In the teachers' report, this relationship was seen with 78.9% of the families accompanying the activities.

The routine did not change between the two moments of collection, changes in routine and family life were as expected^(22)^, which can be explained by the fact that data collection started two months after the social distancing measures were adopted in the municipality in question. In the variable stability in family life, which surveys the times that the family usually spends time together, it was found that at almost all times the family was together, because they were spending more time at home due to social distancing. Changes in the family environment generate instabilities, directly affecting socialization (decreased contact between peers) and economics (decreased income)^(22)^.

Regarding reading at home, there was an increase in students who dedicated themselves to reading in M2, and it can be reasoned that reading increased because it was a time when all the students had online access and classes, which gave them greater motivation to read. Additionally during this period it was easier for the students to read because of the availability of free online books which were made available through platforms such as Google Classroom.

Most parents/guardians said that they talked about school with their children even with the closures, probably because parents needed to supervise school activities more closely, whether it be teaching homework, collecting material from school or accompanying children in online classes, additionally they talked more about television. A study carried out with Brazilians aged 18 and over found that the average time spent watching television was longer after the COVID-19 pandemic, with 1 hour and 45 minutes more viewing time^(23)^. Because families spent more time at home they correspondingly spent more time watching and talking about TV.

When comparing the results of the SDQ-Por^(7,24)^, we can see in Table 3 that there were no differences between M1 and M2 with emotional problems, behavioral problems, hyperactivity, problems with peers, prosocial behavior and the impact and total score of the test. However, a high number of children showed altered or borderline performance on the SDQ, which is concerning. It is important to emphasize that we do not have data on schoolchildren before social isolation and we cannot say that the changes found were due to the pandemic. However emotional issues are presented as a risk factor for children´s development and need to be monitored. A study carried out with Brazilian parents and teachers of 2nd, 3rd and 4th grade students (N=74) showed that 44% of these students had an altered level of emotional problems and 40% of hyperactivity. This study also showed that complaints were more frequently reported in 2^nd^-grade students (N=25), indicating an absence or difficulty in imposing limits on child development^(24)^. It is important to emphasize that reports of emotional problems in the family environment were already common before the pandemic^(25)^, however during social distancing these reports became more evident.

Regarding behavioral problems, three students had an altered level (15.8%), corroborating a previous study with students with an average age of 8.18 years old^(26)^. This data lead to the hypothesis that they may be related to changes in routines, sometimes caused by disobedience and irritability, or the prevalence in this age group of a low socioeconomic level^(27)^.

The instrument's total score is the sum of the scores of 4 scales that refer to difficulties (emotion, hyperactivity, behavioral problems and peer relationships) and the 01 scale that refers to capabilities (prosocial behavior), found in able 3. No difference was found between the two moments, but 17 of the 19 students showed changes in M1, and in M2 this number dropped to 12. This event may have occurred because families and children were already used to the new reality that had been imposed on them.

A study with the same objective carried out with 6,727 adolescents aged 11 to 17 years old reached a total score of 8.8% on the altered scale. In this same sample it was found that students with a low socioeconomic status had higher rates on all the scales of the SDQ^(28)^. Due to social isolation family income was affected which may have led to instability in the mental health of families, and consequently of the children.

It is important to emphasize that the SDQ cannot be seen in isolation for pathological signs, it needs other assessments to complete it. It should be used as a screening for prevention strategies and new adaptations for children^(24)^.

Data taken from the teacher's assessment showed that 18 schoolchildren carried out their activities fully, effectively or partially. One of the objectives of school activities is to consolidate what was taught by the teacher and to measure what was learned in class by them. Many students may not have developed or their knowledge was not measured through reading, writing, text interpretation and comprehension, word ordering and text retelling activities, since in most school reports we did not find answers as to whether the student would be able or not according to the measurement of development. It is worth highlighting the difficulties the education system faced by teachers in evaluating students remotely, often needing to reinvent themselves as a matter of urgency to meet the demands that were imposed on them during the pandemic^(1)^.

When we think about correlating the findings of the two questionnaires (Quest., COVID-19 and RAF), the intention is that they should show us behavior of these students, as well as adjustments so that they can be worked on in a clinical and educational context. We also intended to understand whether having more resources in the family environment associated with pre-established routines lead children to adapt better to social isolation.

Understanding of social distancing is positively related to the number of toys in M2. This may have happened because as children were given toys, which was a positive factor they began to understand the need for social isolation.

Leisure is also positively related to book collections on various subjects. During social distancing students spent more leisure time reading in M1. This data was also found in a study using questionnaires with university students in Macapá - AP. 456 questionnaires on leisure and the impact of the COVID-19 pandemic were analyzed, and reading was one of the leisure activities significantly marked (41.9%)^(29)^.

General outings were positively related in M1 with understanding of social distancing. We can hypothesize that since schoolchildren understood about social distancing, they understood that they needed to stay at home longer and reduce outings and take preventive measures.

There was also a positive relationship in extracurricular activities and an impact on parents' lives. This relationship may have been brought about by the schoolchildren's routines which were divided into moments with the family and activities outside the home. Parents had to take on many new activities to occupy their children's time and this may have had a negative impact on their lives^(22)^. There was also a positive relationship in the routine, including an explanation of preventive measures to which the whole family were committed to caring for themselves and others. Before the pandemic families had routines but during the pandemic, it was necessary to include new information in the daily lives of these students, which resulted in a positive relationship.

Family stability refers to the times when the family is usually together. Here there was a positive relationship, since the family spent more time together, knowledge about preventative measures increased since more time was devoted to explanations and perhaps if the family spent less time together preventative knowledge would have been less as well.

Another limitation of this research concerns the other relevant aspects related to learning, such as reading, writing and comprehension of texts. Although important, they were not part of the scope of this study and should be taken into account in this context when returning to in-person classes.

## CONCLUSION

In view of the findings presented and the changes brought about by social distancing during the attempt to combat the COVID-19 pandemic, these changes affected various areas in the families monitored in this study during the period from June 2020 to December 2020. There was a negative impact on parent’s lives due to the suspension of in-person classes and extracurricular activities and parents had to adapt to new responsibilities and roles. The positive side was that there was an improvement in the family-school relationship, which should be explored further in the post-pandemic period.

Negative changes in routines were also found through the SDQ-Por, causing instabilities in children's mental health.

We propose that school-age children should be monitored, evaluated and given the opportunity of early multidisciplinary intervention, because despite having a less representative sample the study showed us the real situation of 19 families with negative factors that will be reflected in the school development of these children. This study points out as a limitation the description of the progress reports by teachers on school learning, since little more than 60% of the sample did not have this information. We suggest for future research a collection with a more representative sample and a comparison with children from other school years. We also suggest associating the assessment of the questionnaires with the reading performance of students from different socioeconomic levels.
