# Supplementary material for: The learning performance and behavior of school children during the COVID-19 pandemic school closures: from the perspectives of parents and teachers
Source: Codas. 2023 Aug 4;35(4):e20220037. doi: 10.1590/2317-1782/20232022037en (PMC10446755; doi:10.1590/2317-1782/20232022037en)
Supplement: Translation

# Translation: Aprendizagem e comportamento de crianças durante o fechamento das escolas devido à COVID-19: perspectivas dos pais e professores

**DOI:** 10.1590/2317-1782/20232022037pt

**Authors:** Greicyane Marcos de Castro, Vanessa de Oliveira Martins-Reis, Letícia Correa Celeste

**Affiliations:** 1 Universidade de Brasília - UNB - Brasília (DF), Brasil.

**Keywords:** COVID-19, Fonoaudiologia, Percepção, Criança, Ensino, Vulnerabilidade Social, Saúde Mental, Risco, Aprendizagem

## Abstract

**Objetivo:**

Verificar a associação entre a percepção dos cuidadores e professores acerca das mudanças impostas pelo isolamento social e o impacto na aprendizagem de estudantes.

**Método:**

Trata-se de um estudo observacional analítico e longitudinal, com participação de 19 cuidadores (familiares) dos escolares do 2º ano do ensino fundamental de uma escola de financiamento público e seus respectivos professores. Os cuidadores foram submetidos a entrevistas por meio dos questionários: Questionário de Capacidades e Dificuldades, Inventário de Recursos do Ambiente Familiar e Questionário de monitoramento COVID-19 que verifica o comportamento dos escolares e famílias. A coleta aconteceu em dois momentos por ligação telefônica: o primeiro momento (M1) foi iniciado em junho de 2020, e o segundo, (M2) em dezembro de 2020. Os relatórios evolutivos elaborados pelos professores regentes foram recebidos em janeiro de 2021.

**Resultados:**

Foi observada uma implicação negativa na mudança de rotina e um impacto na vida dos pais. Na percepção dos pais, questões significativas e negativas também estiveram presentes na saúde mental das crianças, como por exemplo, mudanças na rotina. Os relatórios de avaliação dos professores mostraram um padrão similar, com a grande maioria deles sem informação sobre o desempenho da aprendizagem. **Conclusão:** Este estudo apontou a importância de acompanhar escolares e famílias em período de distanciamento social, para que possa orientar e intervir juntamente com pais e professores

## INTRODUÇÃO

O distanciamento social foi adotado em diferentes estados do Brasil como medida preventiva de combate à COVID-19, incluindo o fechamento de escolas (Decreto nº 40.550 de 23 de março de 2020 do Distrito Federal). A escola exerce um papel importante no desenvolvimento da criança, e com seu fechamento, as aulas precisaram ser adaptadas à modalidade remota para que o ensino não fosse interrompido. Com isso, veio a necessidade de se traçarem estratégias que auxiliassem na diminuição dos impactos provocados pela suspensão das aulas presenciais^(1)^.

O processo de inclusão digital na educação brasileira já vinha sofrendo desafios e impasses, como dificuldades de acesso à internet na sala de aula, capacitação dos professores no planejamento de atividades, entre outros^(2)^. Considerando tais dificuldades e limitações encontradas, é importante refletir sobre os impactos gerados no processo de aprendizagem da leitura e escrita nesse período de ensino remoto, principalmente em crianças no início do Ensino Fundamental.

Quando comparamos o ensino presencial com o ensino remoto, é preciso levar em consideração questões ou desafios, sejam do ponto de vista do professor, das crianças, das famílias ou das tecnologias. O professor precisou se adaptar ao uso de tecnologias em seu dia a dia profissional para que houvesse interação com os estudantes, elaboração de vídeos, questionários, entre outros. Em relação às famílias, o principal desafio foi a disponibilidade e consciência dos responsáveis para acompanhar as aulas ou explicar o conteúdo para as crianças, sendo que nem sempre tinham escolarização suficiente para isso. As crianças precisavam de motivação para manter a atenção e o ritmo de estudo, e para isso, o desenvolvimento prévio de habilidades e conteúdos acadêmicos foi fundamental. Já quanto à tecnologia, podemos pensar no acesso e domínio de equipamentos e na qualidade da conexão. Essas questões estão fortemente associadas ao nível socioeconômico, pois famílias de baixo nível socioeconômico não conseguem ter acesso suficiente às tecnologias e os responsáveis tendem a ter menor escolaridade^(3)^. Tais questões nos levam a questionar se as condições de acesso que englobam o processo de escolarização online estão sendo monitoradas.

Sabe-se que o desenvolvimento cognitivo e linguístico está associado ao nível socioeconômico, com pior desempenho entre as crianças de baixa renda^(4)^. Dessa forma, destaca-se a importância de acompanhar escolares em situação de vulnerabilidade, pois o acesso à aprendizagem escolar é, por vezes, um caminho maior a percorrer, tornando-se ainda mais importante no contexto de ensino remoto e retorno do ensino presencial. A partir do momento em que se tem conhecimento de como eles estão se desenvolvendo, estratégias podem ser utilizadas para minimizar os impactos causados às famílias.

A literatura aponta que a saúde mental e os recursos do ambiente familiar também estão associados ao desempenho acadêmico de crianças em idade escolar^(5-7)^. Estudo realizado em São Paulo com escolares do 1º ao 5º ano evidenciou que escolares que possuem pior desempenho escolar na visão dos responsáveis são os que mais apresentam problemas relacionados à conduta e hiperatividade^(8)^.

Além dos impactos da saúde mental, o ambiente familiar também influencia positivamente o desempenho escolar^(9)^, quando proporciona: interação familiar com rotinas pré-estabelecidas e mais acesso a atividades extras^(10)^; estímulo à leitura e acompanhamento das atividades escolares^(11)^; e relação família-escola^(12)^.

Com o período de distanciamento social, a relação família-escola passou a ficar distante no contexto de sala de aula convencional, porém houve uma maior necessidade de participação e apoio da família durante esse período remoto^(13)^. Além disso, as crianças ao ficarem isoladas socialmente, perderam oportunidades de lazer e interação com pessoas fora do seu núcleo familiar. Entender como essas famílias vivenciaram e vivenciam esse período de confinamento é uma forma de ajudá-las a entender como enfrentar tais dificuldades. Portanto, o objetivo deste trabalho foi verificar a associação entre a percepção dos cuidadores e dos professores acerca das mudanças impostas pelo isolamento social e o impacto na aprendizagem de estudantes do 2º ano do Ensino Fundamental de uma escola pública da região administrativa de Samambaia-Distrito Federal.

## MÉTODO

Trata-se de estudo observacional analítico e longitudinal aprovado pelo Comitê de Ética em Pesquisa da instituição sob parecer nº 3.906.514. Todos os responsáveis assinaram o Termo de Consentimento Livre e Esclarecido (TCLE). Participaram do estudo cuidadores, compostos pelos familiares responsáveis mais próximos às crianças, e professores de 19 escolares do 2º ano do Ensino Fundamental. Para a atender às mudanças no formato da coleta de dados, que passou a ser remota, os pais que concordaram em participar das entrevistas por telefone, ouviram a leitura do TCLE, falaram o nome e “de acordo” como solicitação do Comitê de Ética para a coleta de maneira remota, sendo esta informação gravada.

Este estudo faz parte de um projeto maior baseado no Modelo de Resposta à Intervenção (RTI) que se iniciou em fevereiro de 2020 de forma presencial. Com o fechamento das escolas, foram incluídas para o presente artigo apenas as crianças regularmente matriculadas nas turmas do 2º ano do Ensino Fundamental I de uma escola pública da cidade de Samambaia- Distrito federal e que estavam com os TCLEs assinados antes do fechamento das escolas. Foram excluídas as crianças cujos pais não participaram das duas entrevistas.

Para coleta dos dados, foram utilizados os seguintes instrumentos respondidos pelos responsáveis cuidadores (familiares):

- Questionário de Capacidades e Dificuldades (SDQ-Por)^(7)^: instrumento utilizado internacionalmente para a detecção de problemas relacionados à saúde mental infanto-juvenil. É composto por 25 itens, sendo 10 itens sobre capacidades, 14 sobre dificuldades e um item neutro. Está dividido em cinco subescalas, cada uma com cinco afirmações, a saber: sintomas emocionais, problemas de conduta, hiperatividade, problemas de relacionamento com colegas e comportamento pró-social. A pontuação do questionário seguiu a proposta do próprio instrumento, com a classificação da pontuação de cada escala e da pontuação total em: normal, limítrofe e alterado.
- Inventário de Recursos do Ambiente Familiar (RAF)^(14)^: roteiro com questões fechadas que possibilita o levantamento dos recursos do ambiente familiar que podem contribuir para o aprendizado escolar nos anos do ensino fundamental. É constituído por 10 questões divididas em três domínios: processos proximais (passeios e viagens; interação com os pais; acesso a brinquedos, livros, jornais e revistas; ocupação adequada do tempo), estabilidade na vida familiar (participação da criança em tarefas domésticas; rotina em família) e vínculo família-escola (participação dos pais na vida escolar e reuniões).
- Questionário de monitoramento COVID-19: Elaborado para o presente estudo, conta com 18 questões sobre o comportamento das crianças e famílias detalhadas a seguir: a suspensão das aulas devido à necessidade de isolamento social; renda da família nesse período; como a criança está lidando com o isolamento social e as informações sobre as medidas de prevenção; e a rotina e os comportamentos da criança durante o isolamento. Para cada pergunta, foi utilizada escala likert de até sete pontos. Para análise de cada variável, somou-se a pontuação da escala likert de cada item, com a seguinte pontuação máxima total: pais explicaram sobre as medidas de prevenção para os escolares, 16 pontos; escolares compreendem o distanciamento social, 12 pontos; sabem que as medidas devem ser feitas, 8 pontos; fazem ou aceitam fazer as medidas de prevenção, 16 pontos; atividades de lazer em casa, 10 pontos; impacto da suspensão das aulas na vida dos pais, 12 pontos; mudanças no comportamento dos escolares, 40 pontos; e mudanças na brincadeira, 54 pontos. Quanto mais próximo da pontuação máxima, mais positivos os comportamentos.
- Relatório evolutivo das crianças: Foi analisado o relatório evolutivo individual das crianças elaborado pelo professor regente ao final do ano letivo de 2020.

A coleta de dados foi realizada em três momentos, sendo as duas primeiras de entrevistas com os responsáveis das crianças, e a terceira de recebimento dos relatórios evolutivos. As entrevistas foram realizadas, pela primeira autora, por meio de ligações telefônicas para aplicação dos questionários descritos anteriormente. O primeiro momento (M1) foi iniciado em junho de 2020, e o segundo momento (M2) em dezembro de 2020. Os relatórios evolutivos elaborados pelos professores regentes foram recebidos em janeiro de 2021. Vale ressaltar que as atividades escolares foram retomadas na modalidade de ensino remoto em 13 de julho de 2020 nas escolas públicas. Durante os meses anteriores (abril e junho), período que não foi contabilizada presença, estratégias foram organizadas e aprimoradas buscando o envolvimento dos estudantes por meio de atividades impressas e plataforma on-line.

Os dados foram submetidos à análise estatística descritiva e inferencial com nível de significância de 5%. As análises foram realizadas com o pacote estatístico IBM SPSS (Statistical Package for Social Sciences) na versão 21.0. Os testes utilizados para comparação entre os dois momentos foram: *Mcnemar Bowker,* para medir o comportamento no momento 1 (M1) e momento 2 (M2) de uma mesma amostra para variáveis qualitativas dependentes, e o *Teste dos Postos de Wilcoxon* para variáveis quantitativas dependentes. Para a análise de correlação, foi utilizado o teste de Spearman para uma distribuição não normal_._

## RESULTADOS

A análise dos dados demonstrou que mais de 80% dos respondentes eram mãe/pai ou avó dos escolares. Em relação à suspensão das aulas, 94,7% concordaram com a decisão governamental de suspensão, como medida de enfrentamento imposta pela COVID-19 e 68,4% gostaram ou adoraram a suspensão das aulas pelos mesmos motivos citados acima.

A coleta realizada por meio do Questionário de monitoramento COVID-19 não levantou as mesmas variáveis em M1 e M2. Para o M2, foram coletadas e comparadas as seguintes variáveis: renda familiar, acesso ao auxílio emergencial, mudança no comportamento da criança, acesso à aula remota e horas de estudo. A Tabela 1 apresenta os resultados da estatística descritiva da pontuação obtida em cada variável do Questionário de monitoramento COVID-19 aplicado no M1 e M2. No M2, apenas a variável mudança no comportamento da criança foi levantada novamente, mas não se observou mudança na pontuação do M1 para o M2 (p=0,536). Para análise do RAF no M1 e M2, comparamos as seguintes variáveis: quantidade de brinquedos, acervo de livros escolares, livros de temas variados, passeios gerais realizados, passeios em lugares especiais, atividades extracurriculares, relação família-escola, rotina, estabilidade na vida familiar, brincar na rua, leitura em casa, conversas sobre a escola e assuntos de televisão. Foram observadas diferenças estatisticamente significantes entre os dois momentos apenas para as variáveis: quantidade de brinquedos, passeios gerais, passeios em lugares especiais e relação família-escola(p-valor<0,05) (Tabela2).

**Tabela 1:** Resultado da estatística descritiva da pontuação do Questionário monitoramento COVID-19

	M1			M2	
	f(%)			f(%)	
Acesso ao auxílio emergencial (Sim)	9 (47,39)		7 (36,84)		
Diminuição da renda (Sim)	12 (63,2)		9 (47,4)		
Aula remota (Não)	14 (74)		0 (0,00)		
Quantas horas diárias são usadas nas atividades escolares					
0h	3 (15,8)			1 (5,3)	
Até 1h	12 (63,16)			9 (47,37)	
Mais de 1h	4 (21,05)			9 (47,37)	
	**Média**	**Mediana**	**DP**	**Mínimo**	**Máximo**
Criança compreende o distanciamento social	7,58	8,00	2,795	1	12
Família explicou sobre as medidas de prevenção	15,26	16,00	1,522	10	16
Criança sabe as medidas que devem ser feitas	6,95	8,00	1,433	3	8
Criança faz ou aceita fazer as medidas de prevenção	13,26	14,00	2,306	8	16
Lazer realizado em casa com a criança	6,42	6,00	2,090	1	9
Impacto da suspensão das aulas na rotina dos pais	14,95	15,00	4,478	4	26
Mudanças comportamentais	48,63	49,00	5,540	37	57
Mudanças na Brincadeira	27,84	27,00	3,760	20	37

Os dados foram expressos pela frequência (f) e porcentagem (%)

**Legenda:** M1 = Momento 1; M2 = Momento 2; DP = Desvio padrão

**Tabela 2:** Resultados obtidos no RAF no M1 e no M2

		**Mediana**	**Mínimo**	**Máximo**	**p-valor** 1
Quantidade de brinquedos	M1	12	6	18	**0,017** *
	M2	15	9	17	
Acervo de livros escolares	M1	6	3	7	0,361
	M2	6	2	7	
Livros de temas variados	M1	5	2	6	0,361
	M2	5	1	6	
Passeios gerais realizados	M1	4	0	4	0,020*
	M2	2	0	4	
Passeios em lugares especiais	M1	6	1	9	0,003*
	M2	2	0	11	
Atividades extracurriculares	M1	1	0	6	0,065
	M2	1	0	1	
Relação família-escola	M1	8	4	8	<0,001*
	M2	10	5	10	
Rotina	M1	11	0	16	0,739
	M2	12	6	16	
Estabilidade na vida familiar	M1	5	2	6	0,67
	M2	6	3	6	
		**F**	**%**		**p-valor** 2
Brinca na rua	M1	10	52,63		1
	M2	11	57,89		
Leitura em casa	M1	10	52,63		0,289
	M2	14	73,68		
Conversa sobre a escola	M1	19	100,00		#
	M2	18	94,74		
Conversa sobre assuntos da televisão	M1	16	84,21		1
	M2	16	84,21		

* p<0,05

1 Teste de Mann-Whitney

2 Teste de McNemar-Bowker

Os dados foram expressos pela frequência (f) e porcentagem (%)

**Legenda:** M1 = Momento 1; M2 = Momento 2; RAF = Inventário de Recursos do Ambiente Familiar; # = Não foi possível calcular

A Tabela 3 não aponta diferença estatisticamente significante para o SDQ-Por entre M1 e M2 (p-valor >0,05). Destaca-se o alto número de crianças com escore alterado e limítrofe na pontuação total e em algumas subescalas mostram dificuldades das crianças nos itens estudados.

**Tabela 3:** Análise das Subescalas do Questionário de Capacidades e Dificuldades (SDQ- Por) - M1 e M2 da coleta

		**Momento 1**	**Momento 2**
		**f** **(%)**	**f (%)**
Problemas emocionais	Normal	9 (47,4)	9 (47,4)
	Limítrofe	4 (21,0)	2 (10,5)
	Alterado	7 (36,8)	8 (42,1)
Problemas de conduta	Normal	12 (63,2)	15 (78,9)
	Limítrofe	3 (15,8)	1 (5,3)
	Alterado	4 (21,0)	3 (15,8)
Hiperatividade	Normal	13 (68,4)	15 (78,9)
	Limítrofe	1 (5,3)	0
	Alterado	5 (26,3)	4 (21,0)
Problemas com pares	Normal	13 (68,4)	14 (73,7)
	Limítrofe	3 (15,8)	2 (10,5)
	Alterado	3 (15,8)	3 (15,8)
Comportamento pró-social	Normal	19 (100)	19 (100)
	Limítrofe	0	0
	Alterado	0	0
Impacto	Normal	13 (68,4)	13 (68,4)
	Limítrofe	1 (5,3)	3 (15,8)
	Alterado	5 (26,3)	4 (21,0)
Total	Normal	0	1 (5,3)
	Limítrofe	2 (10,5)	6 (31,6)
	Alterado	17 (89,5)	12 (63,2)

Teste McNemar-Bowker, nível de significância a 5%

Os dados foram expressos pela frequência (f) e porcentagem (%)

**Legenda:** M1 = Momento 1; M2 = Momento 2

Devido às dificuldades enfrentadas pelos professores na avaliação remota, os relatórios evolutivos das crianças nos mostram um padrão similar, com a grande maioria sem informação (com um pouco mais de 60% da amostra sem informação). Para a análise dos relatórios, buscou-se identificar os seguintes elementos sobre o desenvolvimento das crianças: realizou as atividades da escola; a família acompanhou as atividades; leitura coletiva; leitura silenciosa; leitura e interpretação de textos simples e complexos; leitura e interpretação de textos curtos e longos; compreensão e separação de sílabas; ordenação de palavras e frases; escrita de frases simples e complexas; reconto de texto escrito; e discurso oral (Figura 1). Observa-se que 63,2% dos escolares conseguiram realizar suas atividades e 78,9% das famílias fizeram o acompanhamento das atividades, sendo que apenas 5,3% não realizaram as atividades que foram propostas.

**Figura 1:** Relatório de avaliação do professor no final do semestre de 2020

A avaliação dos professores nos trouxe dados de que 36,8% dos 19 escolares estão em desenvolvimento de leitura coletiva e 31,6%, de leitura silenciosa. Quanto à leitura, 36,8% leem textos simples e 31,6% realizam leitura de textos complexos, curtos e longos e interpretam textos simples e complexos, curtos e longos (Figura 1).

Em relação à escrita, 36,8% escrevem frases simples e 31,6% escrevem frases complexas. Na ordenação de palavras por ordem alfabética, apenas 31,6% a realizam, e na ordenação de frases (início, meio e fim), 36,8% dos escolares a realizam; 31,6% compreendem e separam sílabas. Quanto ao reconto de texto escrito, 36,8% dos escolares realizam esse tipo de atividade. No discurso oral realizado pelos escolares, a porcentagem é de 36,8%. Nos níveis da psicogênese avaliado pelos professores, 5,3% dos escolares encontravam-se no nível pré-silábico, 15,8%, no alfabético II e 15,8%, no alfabético III (Figura 1). Os demais professores não informaram no relatório.

Foi realizado um cruzamento entre o RAF e o Questionário de monitoramento COVID-19 nos momentos M1 e M2. Para essa análise, foi utilizada a correlação de Spearman. Como são muitas variáveis, optou-se por apresentar apenas os resultados estatisticamente significativos. De acordo com os resultados apresentados na Tabela 4, as crianças que possuíam mais brinquedos no M2 e realizavam mais passeios no M1 compreenderam melhor o distanciamento social. Explicar sobre as medidas de prevenção para as crianças foi mais evidenciada na rotina no M1. A estabilidade na vida familiar no M2 fez com que a criança soubesse que medidas de prevenção devem ser feitas. Escolares que cumprem medidas de prevenção diminuem mais os passeios gerais e especiais no M2. No lazer, observa-se que os escolares possuem acervo de livros M1 e livros com temas variados M1. No M2, a diminuição das atividades extracurriculares no RAF impactou na rotina dos pais.

**Tabela 4:** Cruzamento entre os questionários (RAF e Quest. COVID-19) no M1 e M2 da coleta

		Compreende o distanciamento social	Explicou sobre as medidas de prevenção	Sabe as medidas que devem ser feitas	Faz ou aceita fazer as medidas de prevenção	Lazer	Impacto Pais
Quantidade de brinquedos - M2	ρ	0,614**					
	p-valor	0,005					
Passeios gerais - M1	ρ	0,510*					
	p-valor	0,026					
Rotina - M1	ρ		0,571*				
	p-valor		0,011				
Acervo de Livros - M1	ρ					0,522*	
	p-valor					0,022	
Livros de temas variados - M1	ρ					0,522*	
	p-valor					0,022	
Estabilidade na vida familiar M2	ρ			0,579**			
	p-valor			0,009			
Passeios gerais - M2	ρ				-0,485*		
	p-valor				0,035		
Passeios em lugares especiais - M2	ρ				-0,548*		
	p-valor				0,015		
Atividades Extracurriculares - M2	ρ						0,515*
	p-valor						0,024

Teste de coeficiente de correlação de Spearman (〉), nível de significância a 5%

** p<0,01;

* p<0,05

**Legenda:** M1 = Momento 1; M2 = Momento 2; RAF = Inventário de Recursos do Ambiente Familiar

## DISCUSSÃO

Este estudo verificou a associação entre as mudanças impostas pelo isolamento social, a aprendizagem e o comportamento dos escolares. Apresentamos resultados de 19 crianças do 2º ano do Ensino Fundamental, na percepção de seus pais e professores, coletados em junho e dezembro de 2020. Os escolares foram avaliados por meio de questionários (percepção dos pais) sobre a situação do fechamento das escolas, período de isolamento social e relatório evolutivo dos professores.

Os dados mostram que as famílias concordaram com as suspensões das aulas por conta da disseminação do vírus, porém nem todos gostaram. Achados similares foram encontrados em uma pesquisa realizada na República Tcheca em que, apesar da falta de tecnologia e das dificuldades para o ensino das atividades escolares em casa, os pais ainda preferem manter o distanciamento social e o ensino remoto em razão da COVID-19^(15)^. Apesar do estudo tratar de jovens, a similaridade com o presente se dá pela análise contextual na percepção de pais.

A maioria das famílias não teve acesso ao programa de auxílio emergencial no M2. Sabe-se que a diminuição da renda é um agravante que já vinha sendo discutido bem antes do período de isolamento social, porém, nesse momento, foi mais intensificada com resultados ainda mais impactantes que dependerão de quanto tempo durará esse isolamento^(16)^_._

Em relação a como as crianças lidaram com o isolamento social, a maioria dos escolares compreenderam o distanciamento social, pois os pais tiveram o cuidado de explicar sobre as medidas de prevenção que devem ser feitas e tomadas quando necessário. A influência dos pais na educação em saúde é muito importante, pois as crianças se sentem mais acolhidas, além de estarem em um ambiente que traz conforto e confiança^(17)^.

Esperava-se mudanças significativas no lazer, brincadeiras, no comportamento das crianças e na vida dos pais após o fechamento das escolas^(18)^, entretanto, os resultados não mostraram isso. Como o início da coleta de dados ocorreu dois meses após o fechamento das escolas, pode-se inferir que as famílias já estavam se adequando às mudanças impostas pela pandemia.

As aulas autorizadas pela Secretaria de Educação (SEE) do município se iniciaram em 10 de fevereiro de 2020, no entanto, foram suspensas no dia 12 de março de 2020 em função da pandemia da COVID-19. O ensino remoto na SEE iniciou em 13 de julho de 2020 e foram observadas algumas dificuldades de acesso às aulas no M1, com mais de 70% dos escolares sem nenhum tipo de acesso à tecnologia. Porém, no M2 da coleta, todos os escolares já estavam com acesso às aulas, dividindo-se entre uma e seis vezes por semana de aula remota, entre 1h e mais de 1h nas atividades escolares.

É importante frisarmos que mesmo os escolares não tendo acesso à aula no M1, seus cuidadores preocuparam-se em dedicar algumas horas de atividades escolares com livros e outras de livre escolha. Observa-se um dado semelhante tanto de horas de estudo como de dedicação dos cuidadores nas tarefas escolares em uma pesquisa realizada na República Tcheca. Esse estudo (N=9.810) obteve um dado de 72% dos escolares que dedicam entre duas a quatro horas de estudo por dia, e 66% dos seus cuidadores auxiliam com metade desse tempo nas atividades escolares^(15)^_._

No período de distanciamento social, os escolares passaram mais tempo em casa do que o costume. Em relação aos recursos do ambiente familiar, a variável brincar na rua não mudou nos dois momentos, porém a quantidade de brinquedos no M2 foi maior, isso pode ter ocorrido pelo fato dos pais investirem em brinquedos para suprir o lazer desses escolares, trazendo um certo grau de conforto no isolamento^(19)^. Porém, os resultados mostram que, nos livros no geral, não foram observadas diferenças nos M1 e M2, na verdade o acervo de livros diminuiu no M2.

Algumas hipóteses podem ser levantadas aqui para justificar essa diminuição, como por exemplo, o acesso gratuito a livros virtuais, mais acessíveis e com campanhas nacionais ou a doações. Entretanto, apesar de termos diferentes estudos sobre a educação infantil no período da pandemia^(20)^, não encontramos artigos acadêmicos que se debruçassem sobre a variação de livros infantis e o período da pandemia no Brasil. Apesar de não ser um objetivo específico do estudo, deve-se levar em conta a limitação de que não foi possível verificar a variabilidade de livros nesse período, questão que faz parte dos instrumentos utilizados.

Com o isolamento social, os passeios em família diminuíram consideravelmente, demonstrando o cuidado que essas famílias tiveram na situação em que o país está vivendo de distanciamento social. As atividades extracurriculares também diminuíram e elas têm como objetivo a socialização do escolar e que no momento de isolamento social foi diminuída, sendo um ponto negativo para o relacionamento entre pares. O brincar, por exemplo, é uma das atividades que mais auxilia a criança no processo de afetividade, desenvolvimento e socialização^(20)^.

A escola tem sempre buscado o engajamento das famílias no processo de aprendizagem dos escolares, visto que a família é a primeira a começar esse processo. A confiança e o comprometimento são relações que a família e a escola precisam estar em harmonia para que haja uma educação continuada. Neste estudo, vimos que as famílias melhoraram esse relacionamento, precisando ter mais contato com a escola pelo fato de muitas vezes acompanharem seus filhos nas aulas remotas^(21)^. No relatório dos professores, esse relacionamento foi evidenciado com 78,9% das famílias acompanhando as atividades.

A rotina não foi alterada entre os dois momentos da coleta, eram esperadas mudanças na rotina e no convívio familiar^(22)^,o que pode ser explicado pela coleta ter se iniciado dois meses após as medidas de distanciamento social implementadas no município em questão. Na variável estabilidade na vida familiar, que levanta os momentos que a família costuma estar reunida, foi observado que em quase todos os momentos a família estava reunida, tendo em vista estarem passando mais tempo em casa por conta do distanciamento social. Mudanças ocorridas no ambiente familiar geram instabilidades, afetando diretamente os aspectos sociais (diminuição do contato entre pares) e econômico (diminuição da renda)^(22)^_._

Em relação à leitura em casa, foi observado um aumento de escolares que se dedicaram à leitura no M2, podendo-se inferir que a leitura aumentou por ter sido o momento em que todos os escolares já estavam com acesso às aulas remotas e se sentiam mais motivados a ler. Durante esse período, também foi observada a facilidade da leitura através de plataformas que disponibilizaram livros de forma gratuita *(google classroom)*.

A maioria dos responsáveis relatou que conversaram sobre a escola com seus filhos mesmo com o fechamento, provavelmente porque os pais precisaram acompanhar mais de perto a vida escolar das crianças, seja ensinando as tarefas, buscando materiais na escola ou acompanhando as crianças nas aulas online. Da mesma forma, conversaram mais sobre assuntos da televisão. Estudo realizado com brasileiros de 18 anos ou mais, observando que o tempo médio em frente à televisão foi maior após a pandemia da COVID-19, com 1:45 minutos a mais de exposição^(23)^. Provavelmente, como as famílias saíram menos de casa e tiveram mais tempo em frente à televisão, conversaram mais sobre o assunto.

Ao comparar os resultados do SDQ-Por^(7,24)^, podemos observar, na tabela 3, que não houve diferença no M1 e M2 de problemas emocionais, problemas de conduta, hiperatividade, problemas com os pares, comportamento pró-social, impacto e pontuação total do teste. Entretanto, um número elevado de crianças apresentou desempenho alterado ou limítrofe no SDQ, o que é preocupante. É importante enfatizar que não temos dados dos escolares antes do isolamento social, não podendo afirmar que as alterações observadas foram provenientes da pandemia. Porém, as questões emocionais são apresentadas como um fator de risco para o desenvolvimento da criança e precisam ser acompanhadas. Um estudo realizado com pais e professores brasileiros de escolares da 2ª, 3ª e 4ª série (N=74), demonstrou que 44% desses escolares apresentaram nível alterado em problemas emocionais e 40% em hiperatividade. Esse estudo também demonstrou que as queixas são mais relatadas em escolares da 2ª série (N=25), apontando uma ausência ou dificuldade em impor limites no desenvolvimento infantil^(24)^. É importante ressaltar que relatos de problemas emocionais no ambiente familiar já eram comuns antes do período de pandemia^(25)^. No entanto, com o período de distanciamento social, os relatos ficaram mais evidentes.

Em relação aos problemas de conduta, três escolares apresentaram nível alterado (15,8%), corroborando estudo anterior com escolares de média de idade de 8,18 anos^(26)^. Esses dados levam a hipótese de que podem estar relacionados às mudanças de rotinas, causadas às vezes por desobediências e irritabilidade, ou à prevalência, nessa faixa etária, de um nível socioeconômico baixo^(27)^.

A pontuação total do instrumento é a soma dos escores de 4 escalas que se referem a dificuldades (emocionais, hiperatividade, problemas de conduta e relacionamento com pares) e 01 escala que se refere a capacidades (comportamento pró-social), observado na tabela 3. Não foi observada diferença nos dois momentos, mas 17 dos 19 escolares apresentaram alteração no M1, e no M2, esse número caiu para 12. Tal acontecimento pode ter ocorrido em razão das famílias e as crianças já estarem acostumadas com a nova realidade imposta.

Nessa mesma perspectiva, observa-se um estudo realizado com 6.727 crianças e adolescentes de 11 a 17 anos, que obtiveram uma pontuação total de 8,8% na escala alterada. Nessa mesma amostra, foi observado que escolares em nível socioeconômico baixo apresentaram índices mais altos em todas as escalas do SDQ^(28)^. Por conta do isolamento social, a renda familiar foi alterada, podendo ter acarretado instabilidade na saúde mental das famílias, consequentemente das crianças.

É importante salientarmos que o SDQ não pode ser visto de forma isolada para sinais patológicos, necessita de outras avaliações para complementação. Ele deverá ser usado como triagem para estratégias de prevenção e novas adaptações para as crianças^(24)^.

Dados retirados da avaliação do professor revelaram que 18 escolares realizaram suas atividades de forma integral, efetiva ou parcial. As atividades escolares têm como um dos objetivos a consolidação do que foi ensinado pelo professor e medir o conhecimento adquirido em aula por elas. Portanto, muitos escolares podem não ter evoluído ou não foram medidos seus conhecimentos pelas atividades de leitura, escrita, interpretação e compreensão de texto, ordenação de palavras e reconto de texto, pois, na maioria dos relatórios escolares, não obtivemos respostas se o escolar estaria apto ou não de acordo com a medição dos conhecimentos. Entretanto, vale destacar as dificuldades do sistema educacional enfrentadas pelos professores na avaliação dos escolares de forma remota, muitas vezes precisando se reinventar em caráter de urgência para suprir as demandas que no período de pandemia foram impostas^(1)^.

Quando pensamos em correlacionar os achados dos dois questionários (Quest., COVID-19 e RAF), pretendemos que eles nos tragam indicadores do comportamento desses escolares, bem como ajustes para que possam ser trabalhados no contexto clínico e educacional. Pretendemos também entender se o fato de ter mais recursos no ambiente familiar associado a rotinas pré-estabelecidas levam a uma melhor adaptação das crianças no período de isolamento social.

A compreensão sobre o distanciamento social está relacionada de forma positiva com a quantidade de brinquedos no M2. Isso pode ter acontecido, pois, à medida que as crianças foram ganhando brinquedos, que é algo bom para eles, foram entendendo que precisam ficar isolados.

O lazer também está relacionado de forma positiva com acervo de livros de variados temas. Com distanciamento social, os escolares dedicaram mais momentos de lazer com os livros no M1. Esse dado também pode ser evidenciado em um estudo com uma população de jovens universitários de Macapá - AP através de questionários. Foram analisados 456 questionários sobre lazer e impactos da pandemia da COVID-19, e a leitura foi uma das atividades de lazer assinaladas de forma significativa (41,9%)^(29)^.

Os passeios gerais foram relacionados de forma positiva no M1 com a compreensão do distanciamento social. Podemos levantar a hipótese de que, à medida que os escolares compreendem sobre o distanciamento social, entendem que precisam ficar mais tempo em casa, diminuindo os passeios e fazendo as medidas de prevenção.

Foi observado também uma relação positiva nas atividades extracurriculares e um impacto na vida dos pais. Essa relação pode ter acontecido, pois a rotina dos escolares dividia-se em momentos com a família e as atividades fora do lar. Os pais precisaram assumir muitas atividades para preencher o tempo dos seus filhos e isso pode ter impactado de forma negativa na vida deles^(22)^_._ Houve também uma relação positiva na rotina, inclusive ao explicar sobre as medidas de prevenção em que toda a família empenhava-se pelo cuidado dos seus e dos outros. Antes da pandemia, a família tinha uma rotina; com a pandemia, foi necessário inserir novas informações no dia a dia desses escolares, o que ocasionou uma relação positiva_._

A estabilidade familiar diz respeito aos momentos em que a família costuma estar reunida. Nesse âmbito, houve uma relação positiva, tendo em vista que, à medida que a família ficava mais tempo reunida, o saber sobre as medidas de prevenção aumentava, pois havia uma dedicação de mais tempo para tal explicação e que se caso esses momentos fossem menores, talvez o saber sobre as medidas de prevenção seria mais resumido, pouco explicados.

Outro limite desta pesquisa diz respeito aos demais aspectos relevantes relacionados à aprendizagem, como leitura, escrita e compreensão de texto. Apesar de importantes, não foram escopo deste estudo e devem ser levados em consideração nesse contexto de retorno às aulas presenciais.

## CONCLUSÃO

Diante das reflexões apresentadas, as mudanças impostas pelo distanciamento social no período de combate à pandemia da COVID-19 atingiram diferentes esferas nas famílias acompanhadas neste estudo durante o período de junho de 2020 a dezembro de 2020. Houve uma implicação negativa na mudança de rotina e um impacto causado na vida dos pais, pois, com a suspensão das aulas presenciais e das atividades extracurriculares, eles precisaram desempenhar novas funções. De forma positiva, observou-se uma melhora na relação família-escola, que deve ser ainda mais explorada no momento pós-pandemia.

As mudanças na rotina também foram observadas, de forma negativa, por meio do SDQ-Por, gerando instabilidades na saúde mental das crianças.

Propomos que crianças em fase escolar sejam acompanhadas, avaliadas e tenham oportunidade de intervenção multiprofissional precocemente, pois, apesar de apresentarmos uma amostra menos representativa, o estudo nos mostra a realidade de 19 famílias com fatores negativos que serão refletidos no desenvolvimento escolar dessas crianças. Este estudo aponta como limitação a descrição dos relatórios evolutivos dos professores sobre a aprendizagem escolar, uma vez que um pouco mais de 60% da amostra está sem informação nesse quesito. Sugerimos, para pesquisas futuras, uma coleta com amostra mais representativa e uma comparação com crianças de outros anos escolares. Também sugerimos associar a avaliação dos questionários com o desempenho de leitura em escolares de diferentes níveis socioeconômicos.
